# Empty polyetheretherketone (PEEK) cages in anterior cervical diskectomy and fusion (ACDF) show slow radiographic fusion that reduces clinical improvement: results from the prospective multicenter “PIERCE-PEEK” study

**DOI:** 10.1186/s13037-017-0128-y

**Published:** 2017-04-28

**Authors:** Olaf Suess, Martin Schomaker, Mario Cabraja, Marco Danne, Theodoros Kombos, Michael Hanna

**Affiliations:** 1 Spine and Neurotrauma Center, DRK Kliniken Berlin Westend, Spandauer Damm 130, Berlin, Germany; 2 Department of Neurosurgery, Charité University Hosptial, Berlin, Germany; 3 Spine Center, Vivantes AVK, Berlin, Germany; 4 Department of Neurosurgery, Unfallkrankenhaus Marzahn, Berlin, Germany; 5 Department of Neurosurgery, Schloßpark Klinik, Berlin, Germany; 6 Lemessos Medical Center, Limassol, Cyprus; 7 Mercury Spine Healthcare Consulting, New York, NY USA

**Keywords:** Radiculopathy, Myelopathy, Cervical spine surgery, Anterior cervical diskectomy and fusion (ACDF), Clinical trial, PEEK, “Shell” cages, Radiographic fusion

## Abstract

**Background:**

Anterior cervical diskectomy and fusion (ACDF) is a well-established surgical treatment for radiculopathy and myelopathy. Previous studies showed that empty PEEK cages have lower radiographic fusion rates, but the clinical relevance remains unclear. This paper’s aim is to provide high-quality evidence on the outcomes of ACDF with empty PEEK cages and on the relevance of radiographic fusion for clinical outcomes.

**Methods:**

This large prospective multicenter clinical trial performed single-level ACDF with empty PEEK cages on patients with cervical radiculopathy or myelopathy. The main clinical outcomes were VAS (0–10) for pain and NDI (0–100) for functioning. Radiographic fusion was evaluated by two investigators for three different aspects.

**Results:**

The median (range) improvement of the VAS pain score was: 3 (1–6) at 6 months, 3 (2–8) at 12 months, and 4 (2–8) at 18 months. The median (range) improvement of the NDI score was: 12 (2–34) at 6 months, 18 (4–46) at 12 months, and 22 (2–44) at 18 months. Complete radiographic fusion was reached by 126 patients (43%) at 6 months, 214 patients (73%) at 12 months, and 241 patients (83%) at 18 months. Radiographic fusion was a highly significant (*p* < 0.001) predictor of the improvement of VAS and NDI scores.

**Conclusion:**

This study provides strong evidence that ACDF is effective treatment, but the overall rate of radiographic fusion with empty PEEK cages is slow and insufficient. Lack of complete radiographic fusion leads to less improvement of pain and disability. We recommend against using empty uncoated pure PEEK cages in ACDF.

**Trial registration:**

ISRCTN42774128. Retrospectively registered 14 April 2009.

## Background

Anterior cervical diskectomy and fusion (ACDF) is an established standard surgical treatment for radiculopathy [1–3] and myelopathy [1, 4, 5] attributed to degenerative changes of the cervical spine. A recent large metaanalysis of four FDA IDE trials reported an overall 2-year success rate of 70.8% for the ACDF control groups [6]. Although in the past it was not uncommon to perform only ACD with bone graft or even no replacement of the removed tissue at all, this is rarely done anymore; the standard today is to replace the removed disk with a spacer with or without additional bone graft or other materials, in order to achieve bony fusion of the adjoining two vertebral bodies. The purpose of inserting the spacer is to maintain disk height and lordosis, and provide stability, until bony fusion of the two vertebrae can occur to eliminate potentially painful segmental motion. A large metaanalysis has reported that the rate of radiographic fusion for single-level ACDF at an average follow-up of 12 or more months is 92.1% [7]. The implants used in ACDF can be made of several different types of material, including among others: titanium, carbon fiber, stainless steel, polymethylmethacrylate (PMMA), carbonium, or polyetheretherketone (PEEK).

PEEK implants have become a popular choice during the past decade [8]. PEEK has several advantageous properties that explain its popularity. First, PEEK is radiolucent, so x-rays of the spine remain clearly visible [9–14]. Second, PEEK has an elastic modulus similar to that of human bone [9, 14], which may reduce the likelihood of implant subsidence into the vertebrae [15–17]. Third, PEEK is highly biocompatible and durable, making it suitable as a long-term implant [18]. Fourth, PEEK is highly resistant to gamma and electron beam radiation [18], making it safe to sterilize before surgery and to image postoperatively. However, all these material advantages of PEEK would be worthless if the surgery did not help the patient clinically because bony fusion was not achieved.

Several, mostly small, clinical studies reporting on PEEK cages filled with a variety of materials (iliac bone, allograft, DBM, etc.) have reported high rates (90–100%) of radiographic fusion [10–14, 19–23], while a few have reported slightly lower rates (75–89%) [24–29] or remain unclear [30]. Yet in the four previous studies reporting on ACDF with empty PEEK cages, the rates of radiographic fusion have generally been noticeably lower: 88% [15], “30% obvious fusion, 46% probable fusion” at 6 months [31], 72% of levels [32], and 65% of levels or 62% of patients [33]. All four of these studies on empty PEEK cages claimed that there was no correlation between clinical outcomes and fusion status, but all of them were probably too small and underpowered to actually determine this. A larger but retrospective study reported an 85% rate of fusion in single-level cases and 95% in multi-level, but did not comment on the clinical relevance [34]. So although it seems clear that empty PEEK cages have suboptimal rates of fusion, the clinical relevance has not yet been elucidated, and empty PEEK cages remain widely in use.

The “PIERCE-PEEK” study—“Prospective International multicenter Evaluation of Radiological and Clinical Effects of stand-alone Polyetheretherketone (PEEK) Intervertebral Spacers for Anterior Cervical Diskectomy and Fusion”—was a large clinical trial designed to assess the clinical effectiveness of a first-generation PEEK cage, within the standard ACDF procedure, without additional instrumentation or substances, as treatment for single-level cervical radiculopathy or myelopathy. The current purpose of this paper is to report those main clinical outcomes, as well as some unexpected and concerning radiographic findings, which we believe are attributable to the PEEK material itself.

## Methods

### Ethics

The study was approved by the Ethics commission of the Charité University Hospital on May 22, 2006. Patients were permitted to join the study only if they provided written informed consent and were considered competent to do so. Trial registry was uncommon at that time, but the “PIERCE-PEEK” trial was entered into the ISRCTN registry on April 14, 2009 with ID# 42774128.

### Study design & setting

The research was designed as a prospective, international, multicenter, single-arm, clinical trial. Enrollment and surgeries took place at a large university hospital and two teaching hospitals in a major European city (Berlin, Germany) and one private hospital in a small city in another European country (Limassol, Cyprus). Enrollment started in September 2006. Follow-up was scheduled for 6, 12, and 18 months post-op. All radiographs were digitalized and analyzed centrally, as described in more detail below.

### Patients

Patients were eligible for enrollment if they were age 18+, had a degenerative condition between C3 and T1 with clinical signs of radiculopathy or myelopathy, and were otherwise indicated for a single-level ACDF. Patients were excluded if they had: pronounced osteoporosis, fracture of any cervical vertebra, tumor in the cervical spine, previous cervical operations, acute spinal infections, systemic infections, known allergy or intolerance of PEEK, or kyphosis or instability / hypermobility of the cervical spine on functional x-rays.

### Surgery

All patients received a standard anterior cervical diskectomy and fusion (ACDF), using the Smith-Robinson technique [35–38] with implantation of a cage (described in detail below) by one of two surgeons at each of the four hospitals. The cages were not filled with any kind of material. Additional instrumentation was not used. Post-operative neck collars were not prescribed. Further details of the surgical procedure can be found elsewhere [39, 40].

### Implant

All patients in this study were implanted with a “Shell” cage (Advanced Medical Technologies (AMT); Nonnweiler, Germany). These cages were made entirely from pure PEEK, except for four vertical pins embedded in the four corners to enable visualization of the cage on x-rays. The cages were available in the following sizes: 4x16, 5x16, 6x16, 7x16, 5x18, 6x18, 7x18 mm. The top and bottom of the cages had a “toothed” form to reduce the chance of expulsion, but otherwise all surfaces were smooth and uncoated. Further description and illustrations have been provided previously [39, 40].

### Clinical measures

Pain was measured with a visual analogue scale (VAS). The VAS used took the form of a 10 cm ruler, with numbers marked from 0 to 10, “no pain” written to the left of 0 and “maximum worst pain” to the right of 10, with the question “how strong is your pain right now” above the scale. Patients were asked to mark the number 0 to 10 on the VAS, and answers were recorded in the databank as whole numbers.

Patient functioning was assessed with a German translation of the Neck Disability Index (NDI), based upon one we had seen at the German Spine Society annual meeting and which we believe was from the same research group that just recently published a validated German translation of the NDI [41]. (Patients at the one center outside of Germany completed all questionnaires in English.) The 10 NDI questions were scored on their scales of 0–5, summed together, and then doubled, to yield a total score on a 0–100 scale.

In addition to the raw scores for VAS and NDI, the improvement (aka “change scores”) on each of these measures at each study follow-up was calculated by subtracting the follow-up score from the pre-op score. Two recent but small studies indicate that a change of 2.9 points or more on the 0–10 VAS scale would be clinically important in the ACDF population [42, 43]. Based on several studies [42–49], the minimum clinically important improvement of the NDI appears to be about 20 points on a 0–100 scale, a value that has the advantage over other numerical thresholds of meaning that the patient improved on average by one response level on each of the 10 items of the NDI.

The overall outcome at 18 months post-op was assessed by the surgeon using Odom’s criteria [50].

### Radiological assessment

Anterior/posterior, lateral, and functional x-rays were taken at each study visit and digitalized. Radiographic fusion on these x-rays was assessed at all follow-up timepoints at the main study center independently by two people: a radiologist and either the lead surgeon or one specific medical research fellow. In cases where the scoring differed between the radiologist and the other evaluator (<10% of all cases), a third assessment was made by the lead surgeon at the study center outside of Germany.

Drawing on previous guidance from the US FDA and the Surgical Interbody Research Group [51–53], fusion was evaluated by considering three criteria: bony bridging, radiolucency at the juncture of the implant and vertebra, and the amount of motion on dynamic x-rays. For the criteria of bony bridging, 0 to 3 points were given, depending on how many of the five sides of the implant (anterior, posterior, lateral left, lateral right, and the central interior space through the empty cage) showed bridging bone all the way from one vertebra to the other on the x-rays: 0 points for none, 1 point for 1 or 2 sides, 2 points for 3 or 4 sides, or 3 points for all 5 sides. For the criteria of radiolucency, 0 to 2 points were given, depending inversely on the number of implant/vertebra junctures affected. For the criteria of stability / motion, 0 points were given if the range of motion was > 3° on flexion / extension x-rays, and 2 points were given otherwise. The subscores from these three criteria were then summed, yielding a total fusion score of 0 to 7.

For further interpretation and analysis, the raw fusion scores were grouped into four grades of fusion: grade 1 (0–2 points) was considered “no fusion”, grade 2 (3–4 points) was considered “instable ankylosis”, grade 3 (5 points) was considered “questionably stable fusion”, and grade 4 (6–7 points) was considered “complete fusion”. When subsequently describing patients dichotomously as either “fused” or “not fused”, only grade 4 (“complete fusion”) was viewed as “fused”.

**Fig. 1:**
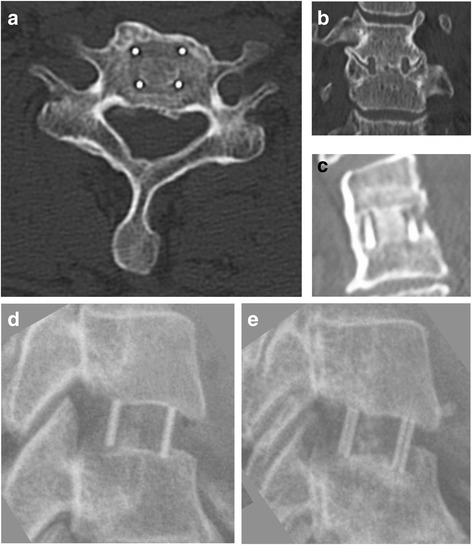
Examples of a good (**a**–**c**) and bad (**d**–**e**) fusion. The example of a good fusion shows CT-scans from C4/C5 of a 56 year-old man at the 12-month follow-up. **a**) Axial view. **b**) Coronal view. **c**) Sagittal reconstruction. The example of the bad fusion shows X-rays of C5/C6 from a 43 year-old woman. **d**) Post-Op control. **e**) At the 6-month follow-up

In the following passages of this report, the “fusion score” refers to the 0–7 raw score; the “fusion grade” refers to the set of four categories, and “complete fusion” or “fused” refers to patients with the grade 4 of fusion (i.e. raw fusion scores 6 and 7), while all other patients have “incomplete fusion” or are “not fused”. Examples of a good and bad fusion are shown in Fig. 1.

As will be briefly discussed below, the present report provides good evidence that this system of scoring radiographic fusion is clinically relevant and methodologically appropriate.

### Statistical analysis

The study sample was characterized and the radiographic and clinical outcomes were reported using descriptive summary statistics and data visualization. The correlation between the improvement in VAS and the improvement in NDI at each of the three follow-up timepoints was calculated using Pearson’s product moment. The correlation between the Odom’s criteria on the one hand and the final VAS score, the final NDI score, or the fusion grade at each of the three follow-up timepoints on the other hand was calculated with Spearman's rank order, because the Odom’s criteria is an ordinal variable. Forward stepwise regression analysis was used to determine if the improvements in VAS or NDI at each of the three follow-up timepoints were dependent upon patient sex, age, operated spinal level, or the fusion score at either the same or a prior follow-up timepoint, with the F-to-enter set at 4.0 and F-to-remove set at 3.9. After seeing the results of these regression analyses, we decided to compare the improvements in VAS and NDI at all follow-up timepoints (as well as the pre-operative VAS and NDI scores) for patients who had versus had not reached complete radiographic fusion at the same or prior follow-up timepoints, using the Mann-Whitney rank sum test, because the data was not normally distributed. To reduce the chance of emphasizing spurious results, we considered results to be statistically significant only at p ≤ 0.01 and highly significant at p ≤ 0.001, while results were only dismissed as “not significant” if *p* > 0.1. All statistical analysis and graphing was performed in SigmaPlot 11.0 (Systat Software; San Jose, CA, USA).

## Results

### Patient enrollment and characteristics

**Fig. 2:**
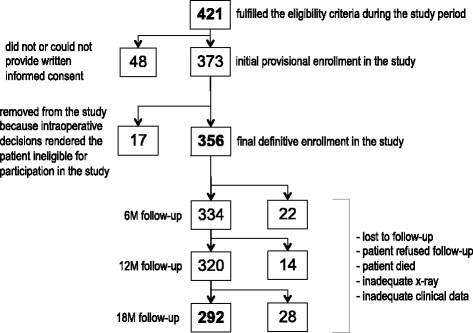
Flowchart of patient enrollment

During the timeperiod of the study, 421 patients fulfilled the study eligibility criteria and 356 of them were definitively enrolled in the study (Fig. 2). Sixty-four patients (18%) were removed from the database because of incomplete follow-up data for various reasons, leaving 292 patients (82%) with complete data for the final statistical analysis presented here.

The study included slightly more men (*n* = 161; 55%) than women (*n* = 131; 45%). The median (range) age was: 54 (31–75). The spinal level operated was C5/C6 for 147 patients (50%), C4/C5 for 72 patients (25%), C6/C7 for 70 patients (24%), C7/T1 for 3 patients (1%), and C3/C4 for none.

### Radiographic fusion

**Fig. 3:**
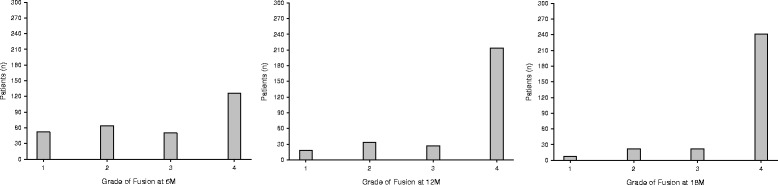
Histograms of the number of patients at the four grades of radiographic fusion. The X-axis shows the four grades of radiographic fusion (as described in more detail in the Methods). The Y-axis is the number of patients. **a**) At the *6-month* follow-up. **b**) At the *12-month* follow-up. **c**) At the *18-month* follow-up

At the 6-month follow-up, 126 patients (43%) had reached complete fusion, while the rest remained distributed among the other grades of incomplete fusion (Fig. 3a). At the 12-month follow-up, 214 patients (73%) had reached complete fusion, while the rest remained distributed among the other grades of incomplete fusion (Fig. 3b). Even at the 18-month follow-up, still only 241 patients (83%) had reached complete radiographic fusion (Fig. 3c).

### Clinical outcomes

**Fig. 4:**
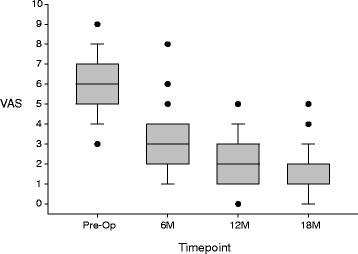
*Box*-and-*whisker plot* of the VAS pain scores. The *X-axis* shows the four data-collection timepoints. The *Y-axis* shows the full range of possible scores of the VAS pain scale. For each of the four box-and-whisker plots, the *middle line* of the box is the median VAS pain score for the study sample, the *top bar* of the box is the 75^th^ percentile, the *bottom bar* of the box is the 25^th^ percentile, the *top whisker* is the 90^th^ percentile, the *bottom whisker* is the 10^th^ percentile, and the dots (if any) are the scores of individual outlier patients outside the 10^th^ to 90^th^ percentiles, but each dot may actually be more than one dot (theoretically, up to 29), overlapping. (Note: where whisker bars are missing, they are equal to the end of the box. Also, the median at the 18-month follow-up was equal to the 25^th^ percentile of 1.)

**Fig. 5:**
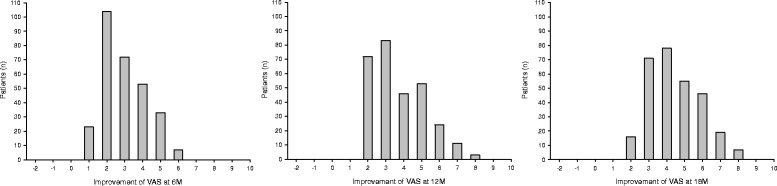
Histogram of the number of patients achieving various degrees of improvement of their VAS pain score. The X-axis shows the amount of improvement in the VAS pain score from pre-up to follow-up, (thus higher numbers are better). The Y-axis is the number of patients with that amount of improvement. **a**) At the *6-month* follow-up. **b**) At the *12-month* follow-up. **c**) At the *18-month* follow-up

Pain levels were moderate to substantial for all patients at pre-op, had decreased to mild to moderate levels by the 6-month follow-up, and settled into the mild range for nearly all patients at later follow-ups (Fig. 4). Clinically important improvements in pain were seen in many patients already by the 6 and 12 month follow-ups and in nearly all patients by the 18 month follow-up (Fig. 5). As can also be seen in the figure, there were no patients whose pre-operative pain scores remained the same or worsened to any follow-up. The median (range) improvement of the VAS pain score was: 3 (1–6) at 6 months, 3 (2–8) at 12 months, and 4 (2–8) at 18 months.

**Fig. 6:**
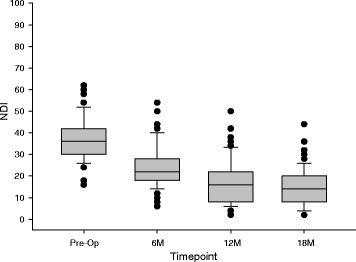
*Box*-and-*whisker plot* of the NDI functioning scores. The *X-axis* shows the four data-collection timepoints. The *Y-axis* shows the full range of possible scores of the NDI scale. For each of the four box-and-whisker plots, the *middle line* of the box is the median NDI score for the study sample, the *top bar* of the box is the 75^th^ percentile, the *bottom bar* of the box is the 25^th^ percentile, the *top whisker* is the 90^th^ percentile, the *bottom whisker* is the 10^th^ percentile, and the dots are the scores of individual outlier patients outside the 10^th^ to 90^th^ percentiles, but each dot may actually be more than one dot (theoretically, up to 29), overlapping

**Fig. 7:**
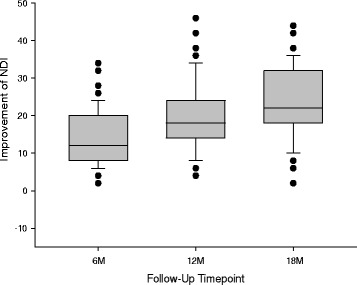
*Box*-and-*whisker plot* of the improvement of their NDI functioning scores. The *X-axis* shows the three follow-up timepoints. The *Y-axis* shows the amount of improvement of the patients’ NDI score from pre-op to follow-up. For each of the four box-and-whisker plots, the *middle line* of the box is the median improvement of the NDI score, the *top bar* of the box is the 75^th^ percentile, the *bottom bar* of the box is the 25^th^ percentile, the *top whisker* is the 90^th^ percentile, the *bottom whisker* is the 10^th^ percentile, and the dots are the scores of individual outlier patients outside the 10^th^ to 90^th^ percentiles, but each dot may actually be more than one dot (theoretically, up to 29), overlapping

Disability was moderate for most patients at pre-op, showed some improvement by the 6-month follow-up, and further gains at later follow-ups (Fig. 6). Very few patients showed clinically important improvement in disability by the 6-month follow-up, but by the 18-month follow-up, a narrow majority of patients did show clinically important improvement of function (Fig. 7). As can be seen in the figure, there were no patients whose pre-operative disability scores remained the same or worsened to any follow-up. The median (range) improvement of the NDI score was: 12 (2–34) at 6 months, 18 (4–46) at 12 months, and 22 (2–44) at 18 months.

At the final follow-up, the Odom’s criteria was “excellent” for 31 patients (10.6%), “good” for 174 patients (59.6%), “fair” for 87 patients (29.8%), and “poor” for none.

### Relationships between clinical outcome measures

As expected, there was a moderate correlation, which was statistically highly significant, between the improvement of the VAS pain score and the improvement of the NDI functioning score at all three follow-up timepoints: *r* = 0.415, *p* < 0.001 at 6 months, *r* = 0.501, *p* < 0.001 at 12 months, and *r* = 0.572, *p* < 0.001 at 18-months. Also as expected, there was a strong and statistically highly significant correlation between the final VAS pain score and the Odom’s criteria (*r* = 0.651, *p* < 0.001) and also between the final NDI functioning score and the Odom’s criteria (*r* = 0.660, *p* < 0.001).

### Relationship of radiographic fusion to clinical outcomes

Sex, age, and spinal level were never significant predictors of the improvement of VAS pain score, and therefore they were not retained in any of these regression models. By contrast, the fusion score at each follow-up timepoint was a predictor of the improvement of the VAS score from pre-op to that same corresponding follow-up, significantly so at 12 months and highly significantly so at 6 and 18 months, though the fusion status alone only accounted for a small amount of the variability in VAS improvement (Table 1). Similar regression analyses on how the fusion score at 6 months predicts the improvement of VAS at 12 and 18 months and how the fusion score at 12 months predicts the improvement of VAS as 18 months yielded similar results that were highly significant using fusion at 6 months and significant using fusion at 12 months (details not shown). Overall, patients who reached complete fusion at each of the three follow-up timepoints showed better improvement of VAS pain scores at that follow-up timepoint and all subsequent follow-up timepoints compared to patients who had not reached complete fusion at that follow-up, and this difference was statistically highly significant for every comparison (Table 2). A similar subgroup analysis found that patients who had reached complete fusion already by the 6 M follow-up (*n* = 126) had statistically significant (*p* = 0.002) better median (25^th^–75^th^ quartile) improvement of VAS pain scores at the 18 M follow-up than patients who had achieved complete fusion only by the 12 M follow-up (i.e. sometime between 6 and 12 months post-op) (*n* = 88): 5 (4–6) vs. 4 (3–5).

**Table 1:** Summary of results for three forward stepwise regression analyses. Each regression analysis retained one significant predictor variable (the fusion score) to predict the improvement of the VAS pain score at the same corresponding follow-up timepoint. The r^2^ indicates the amount of variance of the outcome variable predicted by the model (e.g. model 1 explains 8% of the data variance of the improvement of VAS from pre-op to 6 M). Note also that because each of these models retained only one predictor variable, the standardized coefficient (β) is equal to the correlation coefficient between the predictor variable and outcome variable

Model	Dependent Outcome Variable	*r* ^2^	Constant	Independent Predictor Variable	β	F-to-remove (t^2^)	*p*
1	Δ VAS 6 M	0.08	1.95	Fusion Score 6 M	0.28	25.1	<0.001
2	Δ VAS 12 M	0.03	2.68	Fusion Score 12 M	0.16	7.8	0.006
3	Δ VAS 18 M	0.04	2.87	Fusion Score 18 M	0.20	11.5	<0.001

**Table 2:** Pairwise comparisons of the improvements of VAS pain scores at the follow-up timepoints between patients who were (Y) versus were not (N) completely fused at various follow-up timepoints. There was no statistically significant or clinically meaningful difference of the pre-op VAS pain score when comparing the patients who were versus were not completely fused at each of the three follow-up timepoints (details not shown), meaning that the groups were comparable at baseline. By contrast, all six pairs of comparisons listed in the table were statistically highly significant (*p* < 0.001)

	Complete Fusion					
	6 M		12 M		18 M	
	Y	N	Y	N	Y	N
Δ VAS 6 M: median (25^th^ – 75^th^ quartiles)	3 (2–4)	2 (2–3)	–	–	–	–
Δ VAS 12 M: median (25^th^–75^th^ quartiles)	4 (3–5)	3 (2–4)	4 (3–5)	3 (2–4)	–	–
Δ VAS 18 M: median (25^th^–75^th^ quartile)	5 (4–6)	4 (3–5)	4 (3–6)	4 (3–5)	4 (3–6)	4 (3–4)

Sex, age, and spinal level were never significant predictors of the improvement of the NDI functioning score, and therefore they were not retained in any of these regression models. By contrast, the fusion score at each follow-up timepoint was a statistically highly significant predictor of the improvement of the NDI score from pre-op to that same corresponding follow-up, though the fusion status alone only accounted for a small amount of the variability in NDI improvement (Table 3). Similar regression analyses on how the fusion score at 6 months predicts the improvement of NDI at 12 and 18 months and how the fusion score at 12 months predicts the improvement of NDI as 18 months yielded similar results that were highly significant for all three models (details not shown). Overall, patients who reached complete fusion at each of the three follow-up timepoints showed better improvement of NDI functioning scores at that follow-up timepoint and all subsequent follow-up timepoints compared to patients who had not reached complete fusion at that follow-up, and this difference was statistically significant for one of the six pairwise comparisons and highly significant for the other five comparisons (Table 4). Yet a similar subgroup analysis found no difference in the NDI improvement at 18 M between patients who had reached complete fusion by the 6 M follow-up (*n* = 126) versus patients who had achieved complete fusion only by the 12 M follow-up (i.e. sometime between 6 and 12 months post-op) (*n* = 88).

**Table 3:** Summary of results for three forward stepwise regression analyses. Each regression analysis retained one significant predictor variable (the fusion score) to predict the improvement of the NDI functioning score at the same corresponding follow-up timepoint. The r^2^ indicates the amount of variance of the outcome variable predicted by the model (e.g. model 1 explains 15% of the data variance of the improvement of NDI from pre-op to 6 M). Note also that because each of these models retained only one predictor variable, the standardized coefficient (β) is equal to the correlation coefficient between the predictor variable and outcome variable

Model	Dependent Outcome Variable	*r* ^2^	Constant	Independent Predictor Variable	β	F-to-remove (t^2^)	*p*
1	Δ NDI 6 M	0.15	5.84	Fusion Score 6 M	0.39	51.2	<0.001
2	Δ NDI 12 M	0.18	3.95	Fusion Score 12 M	0.42	62.2	<0.001
3	Δ NDI 18 M	0.11	6.18	Fusion Score 18 M	0.33	34.4	<0.001

**Table 4:** Pairwise comparisons of the improvements of NDI functioning scores at the follow-up timepoints between patients who were (Y) versus were not (N) completely fused at various follow-up timepoints. There was no statistically significant or clinically meaningful difference of the pre-op NDI functioning score when comparing the patients who were versus were not completely fused at each of the three follow-up timepoints (details not shown), meaning that the groups were comparable at baseline. By contrast, all six pairs of comparisons listed in the table were statistically highly significant (all *p* < 0.001, except the comparison of Δ NDI 18 M according to 6 M fusion status was merely significant at *p* = 0.006)

	Complete Fusion					
	6 M		12 M		18 M	
	Y	N	Y	N	Y	N
Δ NDI 6 M: median (25^th^–75^th^ quartiles)	16 (10–22)	10 (8–16)	–	–	–	–
Δ NDI 12 M: median (25^th^–75^th^ quartiles)	21 (16–32)	18 (13.5–22)	22 (16–28)	14 (12–18.5)	–	–
Δ NDI 18 M: median (25^th^–75^th^ quartile)	23 (20–32)	22 (14–30)	24 (19.5–32)	20 (14–26)	24 (20–32)	14 (12–20)

Finally, the Odom’s criteria from the final follow-up showed a moderate correlation with the fusion grade at 6 M (*r* = 0.36), 12 M (*r* = 0.33), and 18 M (*r* = 0.25), and all three of these correlations were statistically highly significant (*p* < 0.001).

## Discussion

The main clinical outcomes of this large prospective multicenter trial confirm that ACDF is effective treatment for patients with cervical radiculopathy or myelopathy, as is already well-known from many previous studies [2–6]. Nearly all patients experienced clinically important improvements in pain by the final follow-up, and no patients had pain that remained the same or worsened post-operatively. The majority of patients also achieved clinically important improvement of disability by the final follow-up. The fact that more patients achieved improvement in pain than disability, and more rapidly, reflects the simple fact that ACDF serves to alleviate pain, while disability takes more time for recovery and often depends on many other factors besides the physical pain in the patient’s neck and arms.

The rate of radiographic fusion in this clinical trial was disappointing. Although the fusion rates shown in the graph (Fig. 3) might not look so bad, it must be kept in mind that really we would like to see 100% of patients with complete radiographic fusion by 12 months, and many previous studies of ACDF have shown that this is possible. But here, only 73% of patients showed complete radiographic fusion by 12 months, and still only 83% of patients by 18 months. This is even below the average rate of 92.1% for single-level ACDF at 12 or more months reported by a metanalysis [7]. Some past clinical trials have reported similar unimpressive numbers, but then interpreted them as great outcomes. But in our viewpoint, if any patient does not achieve radiographic fusion, then the operation has not been entirely successful, from a technical point of view, because one of the two anatomical goals of ACDF is to fuse the vertebrae together, as the letter “F” in “ACDF” implies.

In recent years, it has often been emphasized—rightly so—that the clinical outcomes of pain and disability are the most important criteria to consider, and that achieving radiographic fusion is only of secondary relevance. But it is important to remember that even if a patient is showing improvements in pain and disability (due to the decompression), if he or she is not reaching radiographic fusion, then his or her clinical improvements in pain and disability will probably not be as good as they could have been if radiographic fusion was complete. It is often asserted that radiographic outcomes do not correlate with clinical outcomes [8, 53–55], but we dispute this. That perspective may be based on studies that used poor methods to assess fusion, were too small to detect significant correlations, or used suboptimal or even inappropriate statistical methodology. Our analysis here shows that the degree of radiographic fusion does correlate moderately with the improvement in VAS, to a high level of statistical significance (Table 1), consistent with an earlier analysis [56]. Our analysis also shows that the degree of radiographic fusion correlates even more so, surprisingly, with the improvement in the NDI, to a high level of statistical significance (Table 3), in contradiction to an earlier analysis [56], (which may have suffered here from the methodological limitations mentioned a moment ago). Radiographic fusion does not explain a major amount of the variance in pain and disability outcomes—many other factors are also involved. But radiographic fusion does clearly play a role, and it is one of the few factors that surgeons can easily influence directly. Patients who achieve radiographic fusion show more improvement in pain and disability than patients who remain incompletely fused (Tables 2 and 4). Strikingly, we even found that patients who achieve fusion by 6 months showed more pain improvement at 18 months than patients who achieved fusion by 12 months, thus suggesting that the sooner fusion takes place, the better, even for later pain outcomes.

We believe that the slow and sometimes incomplete radiographic fusion seen in this prospective multicenter clinical trial is due to the use of PEEK as the material for the implanted spacers. Preclinical studies have shown that PEEK is a relatively inert material that does not promote bony ongrowth [18]. A small animal study showed that fusion was achieved at 6 months only when PEEK cages were filled with autolgous iliac graft, not when left empty [57]. Another animal study comparing fusion for PEEK cages versus titanium cages, both with iliac autograft, found slower fusion and less bony ingrowth for the PEEK cages [58]. A paper with a pair of human case reports and an animal model study describes a “halo” effect around PEEK implants on CT-imaging, which reflects lack of bony ongrowth to the implant, even when sufficient bony bridging across and around the implant was clearly apparent [59]. The results of our clinical trial also suggest that PEEK as a material may be better suited for other applications where bony ongrowth is not desired, such as arthroplasty.

The main limitations and strengths of this trial should be discussed to put the reported results into proper perspective. The first limitation is that patients without complete follow-up (64 of 356) were removed entirely from the database. Essentially this means we had an 82% follow-up rate, and no data or analysis on how the lost patients may have differed from the trial completers. Although this is regrettable and not recommended, this kind of complete-case analysis is not uncommon [15, 56, 60–63]. Moreover, a follow-up rate of 80% or better has traditionally been considered adequate in clinical trials [64], and more modern standards for the maximally acceptable missing data [65, 66] have not yet, to our awareness, been proposed for spine surgery. If the loss-to-follow was not at random, the results may be skewed. For example, if all 64 patients removed from the database reached complete fusion by 18 months, then the fusion rate would have been 86% (305 of 356) instead of the reported 83% (241 of 292); whereas, if none of those 64 patients achieved fusion, then the fusion rate would have been 68% (241 of 356). But if the loss-to-follow-up was completely at random, then a complete-case analysis is valid [66] and there is probably no relevant effect on the results reported here. An interesting study on a lumbar degenerative surgery registry found that drop-outs were younger, had a shorter hospital stay, and had a lower rate of surgical complications than completers, but after tracking down nearly all the drop-outs, they also found that there were no significant differences between drop-outs and completers in any of the 2-year clinical outcomes [67]. The second limitation is that the exact forms of the VAS and NDI used were not quite the ideal versions we would select if designing a similar trial today, though they seemed the best available to us at the time. An unnumbered 0–100 VAS scale and a previously validated NDI translation would have been even better, but we do not believe these subtle differences would have changed the results noticeably. Moreover, the VAS and NDI are still the best instruments for assessing pain and disability in this patient population. Third, our clinical trial did not have a comparison group receiving ACDF with an implant made from a different material, so in all scientific stringency, it is not entirely clear how much the inadequate radiographic fusion is due to the PEEK material itself rather than to something else. It is entirely possible that the inadequate fusion is due in part to other factors, such as smoking or osteopenia, (which could also explain lower improvements in VAS and NDI respectively). Nonetheless, our rates of radiographic fusion are lower than what we expected from the literature, and several basic science studies show that PEEK does not favor bony ongrowth, so we believe it is reasonable to conclude that the use of PEEK was the main reason for the disappointing fusion rates.

This study also has several strengths that should be kept in mind. First, this study was a prospective multicenter trial, and thus it provides somewhat higher quality of evidence than most other studies on ACDF. Second, this study had a large sample size, (again much larger than most studies on ACDF in the literature). Despite the removal of patients discussed above, the remaining study sample size was still far above the original target enrollment of *N* = 200 called for in the protocol, based on a power calculation performed by a statistician at our university. The consistent pattern of statistically highly significant results confirms that the study was sufficient powered to provide statistically reliable results. Third, again unlike most studies on ACDF, we used a well-designed and clearly reported system for assessing radiographic fusion, and had two independent researchers assess all radiographs. Indeed, the fact that we found a clear pattern of clinical consequences from incomplete fusion provides strong evidence that the system we used to score the radiographic fusion is clinically relevant and methodologically appropriate: neither too strict, nor too lax, nor likely to be overlooking other relevant aspects of radiographic fusion. We would recommend that other clinicians and researchers also adopt this system of scoring radiographic fusion. A fourth strength of our study is that we found a consistent pattern of results, with high statistical significance and a convincing explanation of the underlying biomedical reasons. We did not need to dredge the data, selectively emphasize cherry-picked results that happened to be marginally significant, and/or engage in speculation about the meaning of the results. The results of our study are clear and coherent, thus implying that they refer to a real clinical issue.

## Conclusions

In conclusion, this clinical trial provides reliable evidence that ACDF is effective treatment for cervical radiculopathy and myelopathy, but it also shows that the use of empty implants made of pure uncoated PEEK is not recommendable. Radiographic fusion occurs only slowly, and sometimes incompletely, when using empty PEEK spacers. Incomplete bony fusion in turn leads to less improvement of pain and disability. If surgeons insist on using PEEK spacers, we recommend also using instrumentation and/or bone graft or other fillers, yet these adjuncts unnecessarily increase healthcare costs and patient safety risks, given the availability of other implants. The first-generation implant used in this study is no longer available on the market. Instead, the current generation of implants use different materials—such as coating the PEEK with other materials (e.g. titanium) or using mixed forms of PEEK (e.g. Carbon-PEEK or Hydroxyapatitit-PEEK). Biomaterials science and basic laboratory studies give us good reason to hope that these next generation implants will achieve better rates of radiographic fusion through better bony on-growth.

## Abbreviations

ACDF: Anterior cervical diskectomy and fusion
DBM: Demineralized bone matrix
NDI: Neck Disability Index
PEEK: Polyetheretherketone
PMMA: Polymethylmethacrylate
VAS: Visual analogue scale
